# Emerging Mechanisms of Physical Exercise Benefits in Adjuvant and Neoadjuvant Cancer Immunotherapy

**DOI:** 10.3390/biomedicines12112528

**Published:** 2024-11-05

**Authors:** Fabio Casciano, Lorenzo Caruso, Enrico Zauli, Arianna Gonelli, Giorgio Zauli, Mauro Vaccarezza

**Affiliations:** 1 Department of Environmental Sciences and Prevention and LTTA Centre, University of Ferrara, 44121 Ferrara, Italy; 2 Department of Environmental Sciences and Prevention, University of Ferrara, 44121 Ferrara, Italy; 3 Department of Translational Medicine, University of Ferrara, 44121 Ferrara, Italy; 4 Research Department, King Khaled Eye Specialistic Hospital, Riyadh 12329, Saudi Arabia; 5 Curtin Medical School, Faculty of Health Sciences, Curtin University, Bentley, WA 6102, Australia

**Keywords:** immune response, immunotherapy, lifestyle, oncology, physical exercise, tumor microenvironment

## Abstract

The primary factors that can be modified in one’s lifestyle are the most influential determinants and significant preventable causes of various types of cancer. Exercise has demonstrated numerous advantages in preventing cancer and aiding in its treatment. However, the precise mechanisms behind these effects are still not fully understood. To contribute to our comprehension of exercise’s impact on cancer immunotherapy and provide recommendations for future research in exercise oncology, we will examine the roles and underlying mechanisms of exercise on immune cells. In addition to reducing the likelihood of developing cancer, exercise can also improve the effectiveness of certain approved anticancer treatments, such as targeted therapy, immunotherapy, and radiotherapy. Exercise is a pivotal modulator of the immune response, and thus, it can play an emerging important role in new immunotherapies. The mechanisms responsible for these effects involve the regulation of intra-tumoral angiogenesis, myokines, adipokines, their associated pathways, cancer metabolism, and anticancer immunity. Our review assesses the potential of physical exercise as an adjuvant/neoadjuvant tool, reducing the burden of cancer relapse, and analyzes emerging molecular mechanisms predicting favorable adjuvanticity effects.

## 1. Introduction

The World Health Organization reports that cancer is the second leading cause of death among noncommunicable diseases [1]. In 2020, there were nearly 19.3 million newly cases diagnosed, with breast cancer being the most prevalent in females and lung cancer being the most prevalent among males [2]. Despite significant advancements in oncology, current anticancer treatments, such as chemotherapy, radiotherapy, endocrine therapy, and surgery, often cause various side effects that impact patients’ daily life and overall well-being. Chemotherapy, a commonly used treatment, has significant implications before and after surgery, affecting morbidity and survivorship [3]. It often reduces cardiorespiratory fitness and muscle strength, leading to decreased functionality, physical independence, and quality of life. Adverse changes in body composition, such as severe weight loss and muscle wasting (cachexia), can also occur. Cancer-related fatigue and poor sleep are prevalent issues among patients undergoing chemotherapy and radiation, greatly impacting their quality of life as well as that of their caregivers [4,5,6,7]. Additionally, endocrine-based therapy causes undesirable effects such as cancer-related fatigue, pain, an increased risk of osteoporosis, alterations in body fat distribution, and impaired cognition, all of which affect physical functioning and overall well-being in patients with cancer [8,9,10].

Exercise is a well-established, safe, and recommended non-pharmacological intervention for all patients undergoing cancer treatment. It offers numerous benefits for overall health and enhances quality of life [11,12,13]. Additionally, patients who engage in higher levels of physical activity experience a significant reduction in the relative risk of cancer mortality and recurrence [12,13]. Animal studies have revealed several biological mechanisms through which exercise can positively impact tumor biology. These mechanisms include the increased expression of pro-apoptotic proteins and tumor suppressor genes, as well as the enhanced infiltration of immune cells within tumors, effectively limiting tumor cell growth and proliferation. Preliminary evidence from human studies also suggests that exercise may decrease the levels of biomarkers associated with tumor proliferation [14,15,16]. Furthermore, exercise plays a crucial role in mitigating the adverse side effects commonly observed in patients undergoing both local and systemic cancer treatments. Aerobic training (AT), resistance training (RT), and combined training have consistently demonstrated positive effects on various health-related outcomes, such as physical fitness, cancer-related fatigue, and overall quality of life [17].

In this light, exercise interventions in the field of oncology are predominantly focused on the adjuvant period after surgery and in survivors, despite recommendations for their implementation throughout the entire cancer continuum, including prevention, treatment, and survivorship [15,16]. As a result, there is a scarcity of high-quality investigations specifically targeting the neoadjuvant phase of cancer treatments [18,19,20]. The neoadjuvant phase, which encompasses the period between diagnosis and surgery, offers a crucial opportunity to assess different exercise benefits in mitigating the treatment’s side effects and improving post-surgery outcomes.

Among interventions in oncology, immunotherapy and manipulation of the immune response have been game-changers in clinical oncology [21,22,23,24]. Immunotherapy has significantly transformed the landscape of cancer care, reshaping our approach to various malignancies. A standout achievement in the realm of immunotherapy is observed in the treatment of melanoma. Previously characterized by limited therapeutic avenues and unfavorable prognoses, melanoma has undergone a remarkable shift with the introduction of immune checkpoint inhibitors, resulting in a substantial enhancement in patient outcomes. Individuals once confronted with a grim diagnosis now benefit from enduring responses and heightened survival rates [24]. Beyond melanoma, immunotherapy has made considerable advancements in addressing diverse cancer types. Notably, immune checkpoint inhibitors have emerged as a standard therapeutic choice in lung cancer, delivering enhanced survival benefits and improved quality of life for patients. Furthermore, promising results have been evidenced in the treatment of kidney cancer, bladder cancer, head and neck cancers, as well as specific lymphomas, among others. Moreover, immunotherapy has broadened its scope of application by incorporating combination strategies. Studies have revealed that the synergistic use of various immunotherapies or the integration of immunotherapy with conventional treatments like chemotherapy or radiation therapy can augment treatment effectiveness [24,25,26,27]. These combined approaches can address multiple pathways implicated in immune suppression and combat cancer cells through diverse mechanisms, ultimately leading to enhanced response rates and prolonged survival outcomes. Therefore, immunotherapy, as a therapeutic tool, can be used by itself and together with other treatments such as chemotherapy, surgery, and radiation.

While advancements in these traditional primary therapies (i.e., immunotherapy, chemotherapy, and radiation therapy) have improved outcomes for many patients, the complexity of cancer and its ability to recur or spread necessitate a multi-faceted approach to treatment. Adjuvant therapy plays a crucial role in this regard, offering additional treatment after primary therapy such as surgery to target any remaining cancer cells that may not be visible in imaging or detectable through other means. This type of therapy is used after the primary treatment to help reduce the risk of cancer recurrence.

Therefore, the main goal of adjuvant therapy is to target any remaining cancer cells that may not have been eliminated by the initial treatment and to prevent the cancer from coming back [28,29,30]. On the other hand, neoadjuvant therapy, also known as preoperative or induction therapy, is a treatment approach increasingly utilized in the management of various types of cancer. Unlike adjuvant therapy, which is administered after surgery to eliminate any remaining cancer cells, neoadjuvant therapy is given before surgery with the aim of shrinking the tumor and potentially making it easier to remove.

This manuscript will explore potential synergies between cancer immunotherapy (adjuvant or neoadjuvant) and physical exercise, focusing on the understudied area of exercise benefits during this therapy rather than delving into the general benefits of exercise for patients with cancer. We will delve into mechanisms that may explain the positive impact of exercise on immune cell activities and discuss the feasibility of applying these mechanisms in adjuvant and neoadjuvant settings.

## 2. Boosting Cancer Immunotherapy with Exercise

Immunotherapy has changed the landscape of human oncology and related therapies. Numerous reviews have covered the overall landscape of cancer immunotherapy and its clinical achievements [31,32,33,34,35,36]. On the other hand, despite its significant beneficial effects, immunotherapy, alone or in combination with chemotherapy or radiotherapy, can induce immune-related adverse events (e.g., autoimmunity) and lead to cancer resistance, primarily driven by the tumor’s multiple and heterogeneous molecular changes [37,38,39].

In this context, exercise-based therapies may enhance the immune system by reducing oxidative stress and associated levels of inflammatory markers. While some evidence supports this notion, there remains controversy and disagreement regarding the extent of this effect [40]. These therapies also contribute to improving physical fitness and are recognized as a significant complementary treatment for various diseases [41]. Table 1 summarizes the main references used in this review that link cancer therapies to their primary effects.

### 2.1. The General Benefits of Exercise on the Immune System and in the Oncology Setting

Engaging in consistent exercise can enhance immune function, reduce the risk of chronic diseases, and promote overall health resilience. Regular physical activity stimulates various physiological processes that bolster the immune system [44,61,73,84]. Moderate exercise has been shown to increase the circulation of immune cells in the body, including lymphocytes and macrophages, which play crucial roles in identifying and combating pathogens. This enhanced circulation allows for a more efficient immune response, enabling the body to detect and respond to infections more swiftly. Moreover, exercise contributes to the reduction in inflammation, a key factor in many chronic diseases [44,53,84]. Chronic inflammation can weaken the immune response and lead to an increased susceptibility to infections. By engaging in moderate aerobic activities, such as walking, jogging, or cycling, individuals can help regulate inflammatory markers in the body. This regulation not only supports immune health but also promotes recovery from illness and injury. Additionally, physical exercise has a profound effect on mental health, which is intrinsically linked to immune function [50]. Regular activity can reduce stress levels and improve mood through the release of endorphins and other neurochemicals. Lower stress levels are associated with a more balanced immune response as chronic stress can lead to immune suppression. Therefore, by incorporating physical exercise into daily routines, individuals can foster both mental and physical resilience against infections. More specifically, one of the primary mechanisms involves the modulation of immune cell populations. Exercise increases the circulation of lymphocytes, including T cells and natural killer (NK) cells, which play crucial roles in identifying and destroying foreign bodies (i.e., pathogens) as well as cancerous cells [64,65]. Enhanced circulation allows these immune cells to more effectively patrol the body and respond to malignant transformations [70]. Moreover, exercise induces a state of mild inflammation that can be beneficial in the context of cancer. This transient inflammatory response stimulates the production of cytokines and other signaling molecules that enhance immune surveillance. The release of these factors not only promotes the proliferation of immune cells but also improves their functionality, enabling a more robust attack against tumor cells [62]. Additionally, physical activity has been associated with improved metabolic health, which is vital for maintaining an effective immune response. Regular exercise helps regulate body weight, reduces adipose tissue inflammation, and improves insulin sensitivity, all of which contribute to a more favorable immune environment. This metabolic balance can diminish the risk of chronic inflammation, a known contributor to cancer progression.

### 2.2. Increased Immune Cell Trafficking, Endothelial Stabilization, and Therapy Sensitization

Exercise has been extensively demonstrated to regulate the cellular immune system by mobilizing cytotoxic immune cells into the circulation through mechanisms involving blood flow-induced shear stress and adrenergic signaling [51,52,60,73,76]. These mobilized cytotoxic immune cells play a crucial role in identifying and eliminating transformed cells. Researchers have shown a significant exercise-induced suppression of tumor growth, potentially attributed to the epinephrine-dependent mobilization of NK cells, leading to increased immune cell infiltration into tumors in mice that engage in wheel-running activities. The administration of the beta-blocker propranolol demonstrated that adrenergic signaling plays a crucial role in inhibiting tumor growth associated with exercise. The intervention with the beta-blocker not only reduced the suppression of tumor growth but also enhanced the mobilization of NK cells and the infiltration of immune cells within the tumors [77]. Although the exercise-induced mobilization of immune cells in patients with cancer is somewhat restricted, research involving breast cancer survivors has convincingly shown their capacity to mobilize NK cells into the bloodstream in a way similar to individuals of the same age who do not have a cancer history [59]. Of note, studies have extensively shown that exercise-induced hyperthermia can impede and slow down tumor growth by boosting the infiltration of NK cells within tumors [63]. An increased body temperature facilitates the movement of immune cells by expanding the diameter of blood vessels within the tumors. Furthermore, apart from this physical impact, an increased body temperature alters the structure of tumor blood vessels by triggering IL-6 trans-signaling, leading to a more conducive environment for cytotoxic T cell movement into tumors [63]. Furthermore, it is worth noting that exercise increases blood flow in tumor vessels, normalizing the endothelial layer and permeabilization (often defective in the tumor ”milieu”), enhancing the possibility of immune cell migration and infiltration into the cancer tissue [54,55].

An intriguing potential mechanism of exercise’s effects involves the enhancement of compromised blood circulation within the tumor microenvironment [54,55,56,85,86]. Compromised blood circulation assists cancer cells in evading immune detection, boosting their ability to spread and metastasize and subjecting them to selective survival pressures that prompt adaptive responses. Normally, immune cells diligently patrol tissues to identify and eliminate pathogens, foreign substances, and abnormal cells [87,88]. However, the hypoxic and acidic conditions in the microenvironment alter the behavior of resident macrophages, which are responsible for recognizing, engulfing, and eliminating dying cells, turning them into pro-tumor and immune-suppressing types. Moreover, hypoxia and acidosis also diminish the effectiveness of immune cells within the tumor microenvironment [89,90,91,92]. Specifically, growth factors and cytokines (such as TGF-β and VEGF) induced by hypoxia or acidosis inhibit the function of T lymphocytes and impair dendritic cell antigen ability to process tumor-related antigens and to present them to lymphocytes [93]. Additionally, hypoxia can directly increase the expression of the immune checkpoint protein PD-L1 by myeloid-derived suppressor cells, dendritic cells, and cancer cells through HIF1α activation, facilitating immune evasion and suppression [43,89,90]. In this light, exercise could counteract these deleterious mechanisms that hamper various immunotherapies.

### 2.3. Positive Modulation of Immune Metabolism

Metabolic byproducts can influence tumor immunogenicity during exercise. Tumors typically exhibit high levels of aerobic glycolysis, leading to the accumulation of lactate within the tumor microenvironment. These elevated lactate levels have the potential to hinder the activity of cytotoxic immune cells, such as T cells [94,95]. However, exercise training has been shown to reduce intra-tumoral lactate levels, which is associated with the regulation of lactate dehydrogenase (LDH) levels [40,80,96,97]. This regulatory mechanism could be clinically significant, as intra-tumoral LDH levels are currently utilized to categorize melanoma patients, with high LDH levels correlating with worse prognoses [81,82,83,96,97]. These findings suggest that the factors promoting immune cell infiltration and mitigating immunosuppressive metabolites can enhance immunogenicity, as observed in exercising mice. Data from studies involving inactive individuals, either with or without a medical condition, show that intense aerobic exercise training leads to enhancements in oxidative phosphorylation and the respiratory capacity of peripheral leukocytes. These improvements were observed across various cell populations, including T cells, neutrophils, and NK cells. It is worth noting that significant respiratory adaptations require a training period exceeding two weeks, as evidenced by a study showing negligible enhancements in PBMC respiration after only a two-week training regimen. These findings resemble skeletal muscle adaptations and highlight the critical role of mitochondrial function in immune response. Furthermore, an improvement in neutrophil and NK cell function has been reported in the context of a likely coordinated adaptation to exercise. Interestingly, recent results suggest that engaging in light to moderate training can positively impact nutrient absorption and maintain mitochondrial balance in peripheral leucocytes [40]. This can potentially enhance the body’s ability to withstand stressors like physical exercise. The research findings from individuals with varying activity levels and health statuses show that light to moderate training may lead to enhanced metabolic performance in various immune cell subpopulations, whether mixed or isolated. It is worth mentioning that light to moderate training in isolated NK cells can produce effects on cell function similar to intensive aerobic exercise training. Participants engaged in light to moderate or high-intensity physical activities showed elevated levels of granzyme B and perforin in their NK cells [40]. In conclusion, current studies indicate that acute exercise can impact the regulation of leukocyte energy metabolism and metabolic function, with training stimuli potentially enhancing metabolic capacity, such as by improving maximal oxidative phosphorylation capability.

### 2.4. Systemic Action of Exercise That Benefits Immune Fitness

Exercise-induced myokines can influence immune cell activity by releasing immune regulatory cytokines, such as IL-6, IL-7, and IL-15 [64]. Various studies have highlighted the significance of IL-15 in stimulating the proliferation, differentiation, and maturation of NK and T cells [65,66]. While less is understood about the role of IL-6 in controlling immune cell function, recent research demonstrated that it promotes the infiltration of immune cells within tumors during exercise. In 2008, Pedersen et al. found that in humans, the levels of IL-6 increase exponentially with physical activity, with the extent of this increase depending on the duration, intensity, and muscle mass involved in the exercise [67]. The administration of anti-IL6 antibodies during exercise blocks IL-6 signaling and reduces exercise-induced tumor growth suppression. Interestingly, it was observed that the direct administration of IL-6 did not replicate this effect, indicating that prior mobilization and priming of immune cells through exercise are necessary for IL-6 to exert its regulatory function.

### 2.5. The Sometimes Contradictory Evidence of In Vivo Studies

The overall picture is promising, and the evidence of a positive effect of physical exercise is mounting. Nevertheless, we must be cautious to reach definitive conclusions, considering that there is a large variability in the reported literature. It is also not easy to compare positive or negative results derived from different scenarios with different exercise programs and in different contexts. Clinical trials have reported that tumor hypoxia and NK cell infiltration are not modified by a single bout of physical exercise in patients with prostate cancer [71,72]; not surprisingly, it is conceivable that an accumulation of several exercise bouts (also considering different training schedules) can impact these outcomes. Future studies are needed to tackle these aspects, keeping in mind new concepts on the effect of hypoxia in the immune system, as well as new methods of harnessing NK cells against cancer tissue [98,99,100].

## 3. Discussion

The emerging mechanisms underlying the beneficial effects on the immune system have an obvious impact on immunotherapy in both adjuvant and neoadjuvant settings. To contextualize the adjuvant/neoadjuvant setting, the positive effects of physical exercise on the immune response against tumor cells need to be linked to the two possible applications of exercise in patients with cancer: “acute” exercise and “regular” exercise.

The mobilization of leucocytes during physical exertion is directly correlated with the level of effort exerted. Elevated blood pressure and shear forces facilitate lymphocyte release from vascular and tissue systems, such as the lungs, liver, and spleen. Consequently, this enhances the migration of leukocytes within the primary blood flow of the peripheral circulation [57,58]. Additionally, the mobilization process is predominantly influenced by adrenaline activating β2-adrenergic receptors present on lymphocytes, prompting their detachment from endothelial surfaces and subsequent re-entry into the bloodstream [78,101].

The precise mechanisms by which exercise and catecholamine signaling influence the mobilization, redistribution, and function of immune cells are not yet fully elucidated. One potential mechanism involves the shedding of the adhesion molecule ICAM-1 from lymphocyte surfaces following stimulation through adrenergic pathways. This process may lead to the detachment of these cells from vascular endothelium in primary (i.e., bone marrow and thymus) and secondary (i.e., spleen and lymph nodes) lymphocyte reservoirs, where norepinephrine and adrenaline are released from nerve terminals or arrive from the bloodstream [102]. Moreover, evidence suggests that β-adrenergic stimulation can trigger the release of IL-6 from skeletal muscle [68]. The response of lymphocyte mobilization during exercise is influenced, in part, by the varying expression levels of β2-adrenergic receptors on different lymphocyte subtypes, with NK cells exhibiting the highest expression, followed by γδ T cells, CD8+ T cells, B cells, and Treg cells [69]. This response is inhibited by non-selective β-antagonists, like nadolol and propranolol, that bind to β2-receptors but not by β1-only blockers such as bisoprolol and metoprolol [79]. Post-exercise, blood lymphocyte counts typically decrease, reaching a nadir within 1–2 h post-exertion. Temporary lymphopenia, where lymphocyte levels drop below pre-exercise values, commonly affects NK and CD8+ T cells but usually returns to baseline within 24 h. This transient lymphopenia is not indicative of immunosuppression and may occur in the context of enhanced immunosurveillance [103]. In healthy individuals, the cytotoxic activity of NK cells against lymphoma and multiple myeloma cell lines can increase by 60% within an hour post-exercise and is accompanied by NK’s receptor expression changes. Animal studies, using fluorescent cell tracking, showed T cells’ redirection from the spleen to target organs like the lungs, as well as the bone marrow and gut following acute physical activity [74]. Furthermore, acute exercise tends to mobilize highly differentiated T cells into circulation, with some exhibiting characteristics associated with exhaustion and terminal differentiation. The ability of acute exercise to rapidly mobilize and increase circulating T cells has been suggested as a strategy to enrich T cell populations in preparation for leukapheresis. Subsequently, these enriched T cells could be utilized for adoptive transfer immunotherapies requiring ex vivo expansion, such as CAR T cells [75].

The enduring positive impacts of consistent daily physical activity may result from the combined effects of repeated short-term exercise sessions and subsequent health-promoting benefits experienced throughout a few hours each day. Each instance of exercise triggers the release of myokines or exerkines and prompts the redistribution of large quantities of lymphocytes [75]. The influence of regular physical activity on overall immune function, specifically on the antitumor immune, response could be associated, at least partially, with the gradual accumulation of frequent short-term episodes of mobilization or the repositioning of active lymphocytes. This process might not manifest as noticeable changes in blood samples or in laboratory tests conducted under resting conditions in individuals following a rest period of at least 24 h after the last exercise session. The immune system’s adaptations to exercise regimens might be more effectively gauged by utilizing dynamic indicators, such as the capacity of NK cells or other immune cell populations to migrate to and infiltrate tumorous tissues [75].

While a single bout of exercise prompts the release of NK cells into the bloodstream, as seen, for example, in individuals with prostate cancer, this singular occurrence is insufficient to enhance the infiltration of NK cells into prostate tissue [72]. However, engaging multiple training sessions over an 8-week period was associated with the increased infiltration of NK cells into prostate tissue in patients with this form of cancer [71]. Furthermore, adhering to the exercise program (up to four days per week) resulted in larger increases in tumorous NK infiltrates by the end of the intervention period (1.60 cells/mm^2^) compared to individuals who did not exercise (0.44 cells/mm^2^) [71]. This discovery holds significance as prostate tumors exploit host macrophages to create a barrier surrounding the tumor, which obstructs the entry of active lymphocytes. Therefore, while a solitary exercise session may not cause alterations in blood-based inflammatory markers among cancer survivors, a cumulative effect from subsequent sessions leads to enhancements.

Exercise training interventions in individuals with cancer or at risk of cancer may not necessarily impact NK cell activity, as demonstrated in vitro with NK cells obtained from blood collected at least one day after the last exercise session [48]. Nevertheless, research indicates a notable increase in NK cell activity following an 8-week aerobic exercise regimen (inclusive of high-intensity interval training) in healthy adults, a 12-week program incorporating high-intensity interval training (HIIT) and RT in patients with chronic lymphocytic leukemia, and an AT and RT protocol conducted during the initial 9–12 weeks of (neo)adjuvant chemotherapy in women with resectable breast or colon cancer [45,46,47]. Finally, regular physical activity or elevated aerobic fitness levels typically achieved through exercise training can also transform the T cell repertoire by diminishing the percentage of dysfunctional and senescent T cells, which have compromised antitumor response capabilities while increasing the proportion of naive CD8+ T cells (CD45RA+CD27+CD62L+CCR7+), which are capable of identifying and responding to novel antigens [49].

To present a balanced view of the field, it is worth noting that exercise may not be universally advantageous for all patients with cancer. Various factors can contribute to potential disadvantages, which warrant careful consideration by healthcare providers and patients alike. While exercise can enhance physical fitness and mental health, certain patients may experience adverse effects that could outweigh these benefits. One significant concern is the risk of injury. Cancer treatments, such as chemotherapy and radiation, can lead to weakened bones, muscle atrophy, and fatigue. Consequently, patients may be more susceptible to strains, sprains, or fractures during physical activity. It is crucial for patients to engage in exercises that are appropriate for their current physical conditions and to seek guidance from healthcare professionals. Moreover, the psychological impact of cancer can complicate a patient’s relationship with exercise. Many individuals may experience anxiety, depression, or a lack of motivation, making it challenging to adhere to an exercise regimen. Additionally, certain types of exercise may exacerbate specific symptoms related to cancer or its treatment. For instance, high-intensity workouts could worsen fatigue or lead to increased pain in patients with certain types of cancer or those undergoing particular treatments. Therefore, it is vital for patients to consult with their healthcare teams to develop a tailored exercise plan that considers their unique circumstances. Of note, in the published literature, the effects of exercise on lymphocyte subpopulations are not univocal and may not be helpful to sustain a valid immune response against cancer. While exercise can offer numerous benefits for patients with cancer, it is essential to acknowledge the potential disadvantages that may arise. By understanding the risks associated with exercise and seeking professional guidance, patients can make informed decisions that prioritize their safety and well-being throughout their cancer journeys.

Focusing more on the mechanistic aspects of immune modulation by exercise, a pivotal future area of study is to ascertain how exercise and hypoxia can impair or complement/synergize with each other while regulating immune system functions. Appropriate acclimatization, training, and nutritional strategies can be used to avoid risks and tap into the synergistic potentials of the poorly studied immune consequences of exercising in a hypoxic state.

Physical exercise has the advantage of being an inexpensive tool to implement in therapy against cancer, and its ability to modulate the immune response against cancerous cells will help in the design of personalized “multi-tool” therapies. Boosting immune response through exercise will undoubtedly lead to a multifactorial approach that does not exclude but will facilitate new innovative therapies, such as new drug delivery approaches and mRNA delivery, and it will pave the way for new combination therapies [104,105,106,107,108].

**Figure 1:**
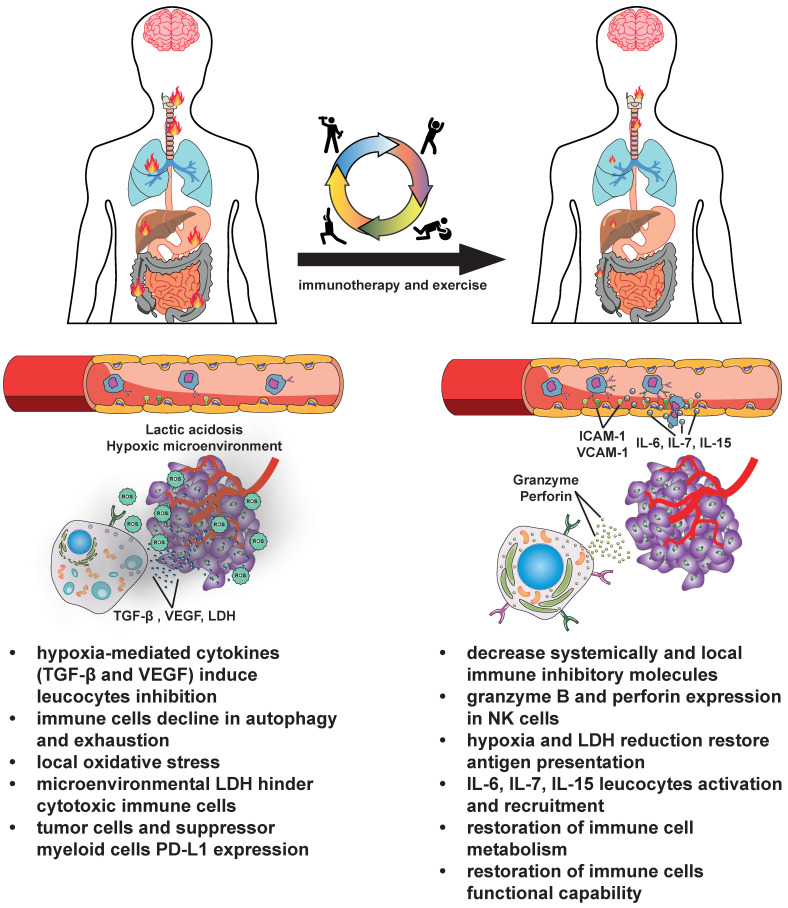
**A schematic representation of the effects of physical exercise.** At baseline (the left part of the figure), cancer cells hinder the immune system through various mechanisms, promoting tumor burden (represented by the fire). Tumor metabolism induces hypoxia, which causes acidosis. This, in turn, reduces antigen presentation by releasing TGF-β and VEGF and the expression of PD-L1 by suppressor myeloid and tumor cells. Additionally, lactic acidosis reprograms the metabolic pathways of immune cells, inhibiting them [109]. Furthermore, elevated blood lactate and local nutrient deprivation impair proper T cell autophagy, reducing their stemness and effector function [110]. In the tumor microenvironment, elevated ROS levels, arising from mitochondria and/or exogenous sources, increase DNA damage and autophagy-mediated tumor cell survival and affect tumor immunity, promoting tumorigenesis [111,112]. Physical exercise can positively engage various mechanisms of the immune system to enhance anticancer therapies, particularly immunotherapies (the right part of the figure). Exercise-induced myokines (e.g., IL-6, IL-7, and IL-15) enhance immune cells, driving their recruitment and maturation at the tumor site [65,66,67]. Furthermore, exercise training shifts the LDH isozyme profile, attenuating the lactate microenvironment in healthy and tumoral tissues [80]. During physical exercise, proper local oxygen concentration is restored, allowing skeletal muscles to drive lactate catalysis. The removal of lactate, in turn, restores immune cell metabolism and their functional capability [40,45,46,47,48,49,81,82,83,113].

A representation of the effect of exercise on improving the immune system capability is shown in Figure 1.

## 4. Conclusions and Future Directions

There is scientific evidence indicating that regular physical activity or exercise can have a positive impact on the immune system by promoting the mobilization of immune cells, potentially aiding their migration to tumors in the hours following each exercise session. Unlike immunotherapies, the immune benefits of exercise do not come with harmful side effects. Tailoring exercise programs to suit the individual characteristics of each patient can significantly improve their health status, including patients in advanced stages of cancer. Experts recommend that all individuals affected by cancer should engage in physical activity to the best of their ability. Further research is required to validate recent preclinical findings and explore the mechanisms through which exercise may prevent tumor formation before it becomes detectable. It appears essential to evaluate the potential effects of exercise as a complementary intervention in immunotherapy trials and in the prehabilitation phase, which involves preoperative interventions to enhance the physiological capacity of patients with cancer for improved surgical outcomes.

We are aware that the evidence provided in our review is not exhaustive: future research will have to better delineate the effect of exercise on the various arms of the immune response, and specifically on the cellular component of the immune response versus cancer. Notably, the most important avenue will likely be to assess the actions of different kinds of exercise (aerobic vs. anaerobic) and exercise schedules on effector T cell exhaustion and their effects on the microscopic tumor–host interface.

More data are needed to better delineate the emerging role of physical exercise as a positive modulator of cancer immunotherapy in the adjuvant/neoadjuvant setting. Further exploration on various exercise modalities, including resistance training, is needed to better understand their impact on anticancer immune function, an area that has received limited research attention compared to endurance exercise.

## Figures and Tables

**Table 1:** Effects of immunotherapy and cancer treatments involving exercise discussed in this review.

**Immunotherapy**	
- High specificity - Remove residual tumor cells - Restore immune function	[24,25,26,27,28,29,30,42]
- Long-term cancer resistance - Severe organ-specific autoimmunity	[28,29,30,43]
**Exercise**	
Enhance leukocyte metabolism and activity	[40,44,45,46,47,48,49]
Increase overall quality of life	[11,12,13,50]
Modulate tumor vessel blood flow	[51,52,53,54,55,56,57,58]
Induce hyperthermia with vessel expansion and tumor growth reduction	[59,60]
Induce release of polarizing cytokines (IL-6, IL-15)	[51,61,62,63,64,65,66,67,68,69]
Mitigate side effects of cancer treatments	[17]
Mobilize immune cells from lymphoid tissues to cancer sites	[70,71,72,73,74,75]
Mobilize immune cells via adrenergic and epinephrine signaling	[59,68,69,76,77,78,79]
Reduce intratumoral lactate	[40,80,81,82,83]

## Data Availability

The data presented in this study are available upon request from the corresponding author.
